# Beneficial Probiotics with New Cancer Therapies for Improved Treatment of Hepatocellular Carcinoma

**DOI:** 10.3390/diseases13040111

**Published:** 2025-04-07

**Authors:** Moeka Nakashima, Akari Fukumoto, Satoru Matsuda

**Affiliations:** Department of Food Science and Nutrition, Nara Women’s University, Kita-Uoya Nishimachi, Nara 630-8506, Japan

**Keywords:** hepatocellular carcinoma, cancer therapy, immune checkpoint, probiotics, gut microbiota, ROS, engram theory, MASLD

## Abstract

Hepatocellular carcinoma (HCC) is a malignant form of primary liver cancer. Intricate networks linked to the host immune system may be associated with the pathogenesis of HCC. A huge amount of interdisciplinary medical information for the treatment of HCC has been accumulated over recent years. For example, advances in new immunotherapy have improved the results of treatment for HCC. This approach can be advantageously combined with standard conventional treatments such as surgical resection to improve the therapeutic effect. However, several toxic effects of treatments may pose a significant threat to human health. Now, a shift in mindset is important for achieving superior cancer therapy, where probiotic therapy may be considered, at least within the bounds of safety. The interplay between the gut microbiota and immune system could affect the efficacy of several anticancer treatments, including of immune checkpoint therapy via the alteration of Th17 cell function against various malignant tumors. Here, some recent anticancer techniques are discussed, whereby the growth of HCC may be effectively and safely repressed by probiotic therapy.

## 1. Introduction

**Figure 1:**
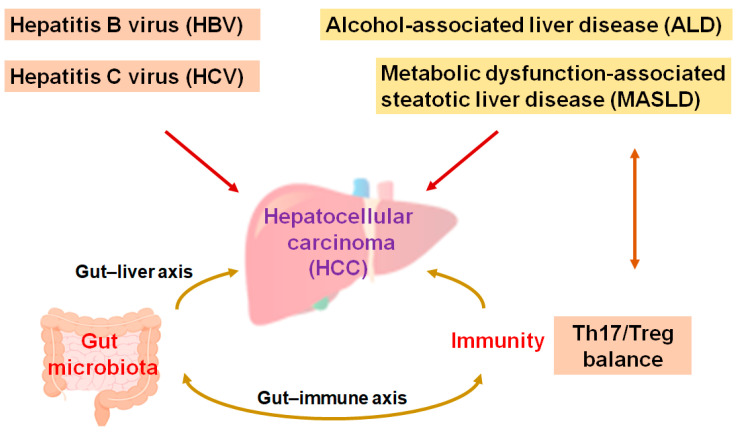
A hypothetical representation and overview of the pathogenesis during the development of hepatocellular carcinoma (HCC). Hepatitis B virus (HBV), hepatitis C virus (HCV), alcohol-associated liver disease (ALD), and/or metabolic dysfunction-associated steatotic liver disease (MASLD)—as well as Th17/Treg balance and/or the gut microbiota—may independently contribute to the pathogenesis of HCC. The arrowhead indicates stimulation whereas double-arrowheads suggest bidirectional stimulation. Note that several important activities such as anti-inflammatory reaction pathways have been omitted for clarity.

The main liver cancers, including hepatocellular carcinoma (HCC), are the second most lethal cancer globally. Cases of HCC approximately constitute three quarters of malignant cases, and HCC is such an aggressive malignancy that it become a social healthcare problem [1]. Medical treatments for HCC may be decided based on the clinical stages of the disease. In general, local therapies such as resection—including hepatectomy, ablation, and/or irradiation—are the traditional treatment for early-stage HCC [2]. Surgical management may be the most vital method for HCC patients to achieve long-term survival. However, the 5-year survival rate might not be satisfactory, with a considerable rate of patients relapsing within 5 years of surgery [3]. In addition, HCC is frequently detected at the point of unresectable advanced stages [4], which may ensure that the cure rate is extremely low [5]. Instinctively, systematic palliative management might be an opportunity for most patients with advanced-stage HCC [6]. Although viral hepatitis, specifically the hepatitis B virus (HBV) and hepatitis C virus (HCV), is one of the most significant causes in the development of HCC, alcohol-associated liver disease (ALD) and/or metabolic dysfunction-associated steatohepatitis (MASLD) may also be noticeable causes in the development of HCC [7]. The ingestion of high fat in addition to bacterial endotoxins is often a probable cause of MASLD and HCC [8]. In addition, liver fibrosis, aflatoxin-induced liver toxicity, diabetes, smoking, and immune-related diseases including autoimmune hepatitis could also be important risk factors for the development of HCC [9,10]. Interestingly, increasing evidence has pointed to an important role of the gut microbiota and/or their various metabolites for the development of HCC. An imbalance in the composition of gut microbiota may result in chronic inflammation and/or the development of MASLD/HCC (Figure 1) [11,12].

As for HCC treatment, resection surgery might generally be the primary method; however, it has a high recurrence rate. Preoperative medical factors such as anesthesia, hypothermia, and systemic inflammatory responses may support the micro-relapse of early cancers [13]. In addition, a problem in the surgical removal of tumors may be the risk of cancer cells detaching and entering systemic circulation [13]. Occult microscopic cancers in different organs, including the prostate and/or breast, are common in the general population [14]. Under these conditions, immunotherapy synergizes with radiotherapy, enhancing their antitumor reactions, with favorable clinical consequences [15]. Subsequently, many patients may exhibit greater effects from the combined radioimmunotherapy [16,17]. Hence, further optimization of the combination might be increasingly helpful [18]. In general, cancer therapy promotes the production of reactive oxygen species (ROS) [19], which can yield some inflammation in non-target organs [20]. Cytotoxic lymphocytes, neutrophils, and/or macrophages may move to the place of inflammation to clear damaged cells [21]. Inflammatory cells may secrete various chemokines, cytokines, and growth factors, which may accidentally promote the cell growth of various tumors [22]. Additionally, adverse reactions to anticancer drugs may occur more severely than for those of other treatment drugs, which might further damage healthy cells. In addition, the treatment effect would be reduced and/or even vanish via the development of drug resistance [23]. Therefore, some arrangement of chemotherapeutic drugs were developed to achieve a more promising strategy and obtaining an improved survival rate [24]. Inventions in technology and operative techniques have been required for further improved survival rates [25]. Particularly, innovative treatments with few side effects are immediately required. Here, we discuss some of the recent anticancer procedures, which could effectively contribute to the inhibition of HCC growth. This paper would also fall into the future concept of cancer treatments with the combination of probiotic ability, which can contribute to the improved efficacy of new cancer therapeutics of HCC.

## 2. Recent Cancer Therapies Possibly Applied for the Treatment of HCC

Photothermal therapy (PTT) and photodynamic therapy (PDT) are relatively new strategies for cancer therapy, which are therapeutic methods with low toxicities utilizing photosensitizers preferentially accumulating in tumor tissue. PTT and PDT have gradually emerged because of their spatial selectivity and/or relatively lower resistance of therapy, which could be utilized in combination with other therapeutic modalities such as chemotherapy and/or immunotherapy. Usually, PTT may use photosensitizers with specific light absorption to convert into a heat energy for eliminating cancer cells with the apoptosis, while PDT can exploit these photosensitizers to yield an adequate amount of ROS by the particular wavelengths of light that also eliminate cancer cells. PTT causes relatively little damage to surrounding healthy cells, since thermal effects only occur when specific light is applied in the presence of photosensitizers [26]. When the tumor tissues are exposed to the specific light, the activated photosensitizers might produce ROS in the usage of PDT, which could also damage tumor cells and/or the neovascularization to tumors [27]. Photosensitizers for PTT and/or PDT may contain several metal materials and carbon-based nanoparticles, which might also produce ROS for the regulation of tumor growth [28]. Again, cancer therapies may promote the production of ROS, which yields inflammation in the non-target organs [19,20]. Prostate cancer is one of the most common cancers in men and is asymptomatic in the early stage of the cancer with favorable indication of PTT/PDT therapies. These PTT/PDT therapies might considerably improve the outcome even in advanced prostate cancer with reduced systemic toxicity. In addition to these therapies, laser-induced PTT against HCC has attracted extensive attention because of its strong tissue penetration with favorable biosafety [29]. Moreover, data have also confirmed the inhibitory effect of PDT in tumor cell growth both in HCC and cholangiocarcinoma [30].

Procedures with immune checkpoint inhibition might be one of the most successful therapies for a number of cancers [31], which may activate specific immune cells such as CD3^+^ T cells via the inhibition of a family of signaling receptors expressed on the surface of lymphocytes [32]. The signaling of immune checkpoints is indispensable in preventing excessive immune responses that could result in damage to the host tissues [33]. Hence, their inhibition has been recognized as a potent therapeutic mechanism in certain tumors [34]. Frequently used immune checkpoint inhibitors against various tumors may be made up of beneficial monoclonal antibodies such as ipilimumab, which can target the immune checkpoint of programmed cell death protein-1 (PD-1) and/or cytotoxic T-lymphocyte-associated protein 4 (CTLA-4) [35]. Immunotherapy with these immune checkpoint inhibitors has been developed for application in patients with a wide range of advanced cancers including Hodgkin’s lymphoma, melanoma, non-small cell lung cancer, and/or head and neck squamous cell carcinoma [36]. It is amazing that the application of immunotherapy could achieve complete responses within approximately 30% of patients [37]. Immune checkpoint inhibitors have been also permitted for clinical usage in HCC treatment, which has revealed considerable efficacy in many clinical trials afterwards [38]. Thus far, programmed cell death ligand 1 (PD-L1), CTLA-4, PD-1, and/or several other immune molecules seem to be connected with their effectiveness for the advanced cancer therapy, also being associated with the modification of the tumor microenvironment [39]. Each treatment could result in the stimulation of an anticancer immune system for killing cancer cells [40]. Favorably, few side effects have been noticed except reversible minor adverse events [41,42,43]. Mechanistically, the physiological association between the PD-1/PD-L1 pathway and both Th17/Treg cells has been shown, which may suggest a crucial role of PD-1/PD-L1 in the regulation of Th17/Treg cells [44]. Likewise, CTLA-4 therapy could also support Th17 cells [45]. In addition, the obstruction of CTLA-4 signaling could also further inhibit the function of Treg cells [46]. Interestingly, IL-17 and Th17 cells could upregulate PD-L1 expression, which may impede the efficacy of the immunotherapy [47,48]. Interaction of PD-1 with PD-L1 might trigger a reduction in PI3K signaling in T-lymphocytes leading to the introduction of Treg cells [49]. Th17 cells can ultimately differentiate into suppressive Treg cells [50], which may provide as a source of tumor-associated Treg cells. Therefore, excessive inflammation with Th17 cells might play imperative roles in some inflammation-associated carcinogenesis [51], also targeting several specific antigens presenting in malignant cancer cells [52]. The strategies to overcome this immune therapy-resistance of cancer cells would move toward an intensified anticancer strategy encompassing a procedure with low adverse events.

With recent advances, chimeric antigen receptor (CAR) T-cell immunotherapy has similarly become a favorable modality for patients with refractory cancers. CAR immunotherapies commonly utilize synthetic constructs that can bind to a specific target antigen in a major histocompatibility complex (MHC) independent manner. In general, the MHC could trigger a strong T-cell activation directing to the removal of target cells. The successful results of CAR-T-cell immune therapy in relapsed and/or refractory B-cell malignancies has shifted the paradigm of this immunotherapy by attracting the medical attention for the treatment of various solid tumors [53,54]. Afterward, the CAR-T-cell immunotherapy has also achieved promising success in the treatment of HCC [55,56]. Identification of more specific targets in HCC might improve the therapeutic potential of the CAR-T-cell therapy in the future [57].

Oncolytic virus therapy characterizes an effectual immunotherapeutic approach against cancers. The oncolytic virus may stimulate antitumor responses both through tumor cell-specific cell lysis and by the activation of certain immune system. Some viruses are naturally capable of killing cancer cells [58]. With the genetic engineering modification, virus mutants with oncolytic activity could be just restricted to tumor cells, which might reduce the virus-induced non-specific toxicity in normal tissues/organs. There is growing evidence that the success of oncolytic virus therapy may depend on the tumor microenvironment [58], which is considered to be the most promising cancer treatment along with surgery, chemotherapy, and/or radiotherapy [59]. Several studies have provided evidence for the application of oncolytic virus therapy in HCC [60,61]. In addition, oncolytic vaccinia virus could significantly enhance the cytotoxicity in HCC through the activation of PI3K/AKT signaling pathways [61].

## 3. A New Concept for the Effective Cancer Therapy

Oncology is one of the most interdisciplinary research fields. In addition, a wide range of diagnostic and/or treatment technologies are accessible in the field. Even though carcinogenesis has been comprehensively studied, a shift in mindset might be required for the concept of forthcoming cancer therapy. The usefulness of treatment procedures against cancers might principally depend on the balance between cure efficacy and toxicity of the therapy. In this respect, probiotics could be used at least as an adjunctive therapy for several cancer treatments [62,63]. Humans and bacteria have a symbiotic relationship, which may sustain substantial influence on our health. Residential bacteria have been identified everywhere in tissues including the brain, kidney, placenta, and breast [64,65]. Interestingly, some bacteria strains have been identified to possess a therapeutic capability for an anticancer activity [64]. Some bacteria could also inhibit the growth of solid tumors. On the contrary, it has been shown that *Helicobacter pylori* has been proved to increase the risk of gastric cancer development [65]. In addition, enterotoxins of *B. fragilis* may contribute to cancer developments through the activation of signal transducer and the activator of transcription (STAT) signaling pathway [66]. Certain commensal bacteria may also inhibit the development and progression of inflammatory bowel disease-related cancer [67]. Similarly, it has been reported that *Saccharomyces boulardii* (*S. boulardii*) can effectively reduce the carcinogenesis in an AOM/DSS-induced mouse model of cancer [68]. In addition, *Clostridium butyricum* can enforce the inhibitory effect of inflammation in mouse intestine [69]. Furthermore, a strain of *Salmonella typhimurium* can trigger cell death in prostate cancer cell lines [70]. *Clostridium novyi* can also cause direct cytotoxicity to cancer cells via the disruption of lipid bilayers [71]. Interestingly, these therapeutic bacteria could constructively colonize in the hypoxic area nearby malignant tumors [72]. It has been shown that the combination of *Salmonella typhimurium* with traditional cancer chemotherapies could prolong the survival rate in mice model [73]. Consequently, many bacteria-based cancer therapies would have proceeded through clinical trials [74]. The application of bacteria-based cancer therapies may also cover several side effects in the treatment of radiotherapy and/or chemotherapy [74]. In particular, certain probiotics could reduce the incidence of cancer therapy-related side effects such as oral mucositis and/or diarrhea [75]. Studies regarding *E. coli*, *Salmonella typhimurium*, and *Salmonella clostridium* strains have demonstrated that bacteria-based cancer therapies combined with radiotherapy can reduce radiation-associated adverse damages [76,77], which can also enhance the therapeutic effect and then the prognosis [78,79]. However, the detailed behavior of association with cancer cells, bacteria, and immune cells during the bacteria-based cancer therapies needs more in-depth investigation. It has been reported that the interrelationship between the immune system and the gut microbiome could even determine the effectiveness of the cancer immunotherapy [80]. Therefore, modulation of the gut microbiome could optimize therapeutic outcomes on the immune-checkpoints blockade therapy. For example, it has been reported that *L. acidophilus* combined with anti-CTLA-4 antibody blockade can enhance the antitumor immunity by synergistically improving antitumor T-cell immunity in mouse model [81]. In addition, the antitumor effects of CTLA-4 blockade may also be determined by *Bacteroides* species including *B. thetaiotaomicron* or *B. fragilis* [82]. Consistently, malignant tumor cells in antibiotic-treated mice had no response to a CTLA-4 blockade therapy, suggesting that the beneficial bacteria may be deceased by the use of antibiotics [82,83]. Also, salvage with the *B. fragilis* could retrieve the effectiveness of CTLA-4 blockade therapy [83]. *F. prausnitzii* has anti-inflammatory properties recognized in colitis model animals [84], in which the butyrate produced from the *F. prausnitzii* could improve the Th17/Treg balance for exhibiting anti-inflammatory effects [85]. In these ways, the relationship between gut microbiota and cancer therapy has intensively been studied for the development of effective cancer therapy [86].

## 4. Epigenetics with Gut Microbiota Alteration Involved in the Superior Cancer Therapy

**Figure 2:**
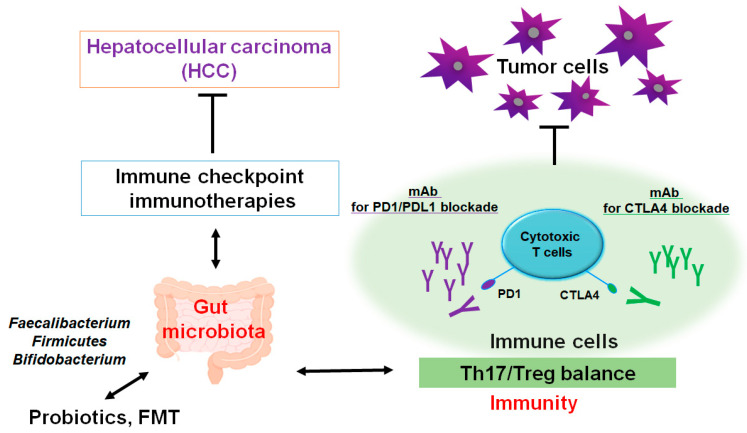
A schematic representation and hypothetical overview of the immune checkpoint inhibitors against HCC. Certain gut microbiota could contribute to the potentiation of the immune checkpoint immunotherapy with the improvement of Th17/Treg immune cells’ balance. Some kinds of probiotics and/or fecal microbiota transplantation (FMT) could contribute to the alteration in gut microbial community for playing valuable roles to the potentiation of immune checkpoint therapy. Examples of certain beneficial microbial species with some effects on anticancer immune responses have been shown. Arrowhead indicates stimulation (or bidirectional stimulation), whereas hammerhead shows inhibition. Note that several important activities such as cytokine or chemokine production pathways have been omitted for clarity.

Evolving evidence has connected a crucial role of gut microbiota in liver inflammation and/or the development of HCC [87]. Undoubtedly, the gut–liver axis might be the vital mechanism by which the gut microbiota could promote various liver diseases including HCC [88]. For instance, dietary cholesterol could induce the alteration of gut bacterial metabolites, which might be involved in the MASLD-associated HCC [89]. In addition, gut microbiota dysfunction may trigger a neutrophil accumulation in the gut epithelium that could change the composition of inflammatory cytokines affecting T helper 17 (Th17) cells [90]. Intra-tumoral areas of HCC might be often governed in an immunosuppressive situation [91], where an amount of regulatory T (Treg) cells could construct a link to the immune escaping [92]. Correspondingly, increased quantities of Th17 cells have been identified in tumor tissue and even in peripheral blood of patients with HCC [93,94], which might be linked to negative outcomes of HCC prognosis [93,95]. Comparable results have also been observed in animal models, whereby limiting expansion of Th17 cells can decrease the growth of transplanted liver tumors in animal model [96]. The gut may be a possible site of Th17 cell production. In addition, gut microbiota might influence the differentiation of Th17 cells via regulating the function of dendritic cells [97]. Accordingly, there may be an intricate association between the development of Th17 cells and the progression of HCC with the roles of gut microbiota (Figure 2).

The resource of tumor-associated immune Th17 cells might be also linked to the function of gut [98]. Furthermore, Th17 cells appear to be associated with the HCC development possibly via facilitating the angiogenesis of tumors [96,99]. Remarkably, modification of the gut microbiota could assist in avoiding the incidence of the HCC via the suppression of Th17 cells [100]. However, the alteration in gut microbiota may coincidentally induce the tumor development. For example, some gut bacterial metabolites have been known to play a role in the carcinogenesis of various tumors [101,102]. In addition, adjustment of gut microbiota reveals the effective procedure of strengthening antitumor immunity, which implies a close link between gut microbiota and the oncopathogenic mechanisms of various tumors [103]. The usage of several methods including probiotics and/or the fecal microbiota transplantation (FMT) may develop new models with the potential expediency available for the HCC therapy. Especially, Th17 cells appear to be an advanced therapeutic target against certain conditions of cancer-promotion such as excess production of ROS and/or severe inflammation. Tactics with employing probiotics and/or FMT could be advantageous in order to slow down the growth of HCC.

Collecting evidence shows the association of the dysbiosis of gut in the HCC development. Some intestinal bacteria isolated from patients with metabolic dysfunction associated steatohepatitis (MASH) may present an increase in *Klebsiella pneumoniae* strains [104]. In addition, the predominant bacteria in patients with cirrhosis-HCC are *Clostridium* and *Paraprevotellaceae* family species [105]. Depletion of these bacteria might prevent the HCC progression. For example, alteration in the gut microbiota by some antibiotic treatments may decrease the liver tumor growth via the mechanism of natural killer T-cell accumulation [106]. Now, the gut microbiome has appeared as an important factor controlling antitumor immunity governing the efficacy of chemo- and/or novel immune-therapies [107,108,109]. The liver may possibly be exposed to bacterial components and their metabolites via the portal vein, in which the gut microbiome could potentially regulate the development of cancers including HCC [110,111]. Therefore, gut commensal bacteria could be potential targets for controlling the liver tumorigenesis. Interestingly, it has been shown that patients with HCC have been identified to possess a higher proportion of *Akkermansia muciniphila* and *Ruminococcaceae* spp. [112]. The function of commensal bacteria might play a key role in preserving the cancer-related immune homeostasis of the host [113].

## 5. Association Between Gut Microbiota and Useful Epigenetics for the Development of Effective Cancer Therapy Against HCC

Epigenetic regulations include a modification in DNA methylation, epigenetic silencing of microRNAs, a histone protein phosphorylation, and a histone acetylation may be involved in various gene expressions [114]. These regulations have also been recognized as playing a substantial role in carcinogenesis as well as in antitumor immunity [115]. Therefore, the application of some epigenetic technologies could be favorable for enhancing the cancer therapy. In fact, it is becoming clear that epigenetics can play a significant role in carcinogenesis for the cancer prevention [116,117,118]. On the other hand, several tumor-related bacteria may lead to cancer initiation and/or development by inducing the epigenetic alterations in the inhibition of host immunity [119]. Some events from gut microbiota have been shown to result in considerable responses by CD4^+^/CD8^+^ T-cell effector subpopulations [120]. In particular, pathogenic bacteria could play a significant role in the dysregulation of the epigenetic machinery for their target cells. Alterations in the gene expression pattern could also interfere with the activity of cancer immune responses [121]. In addition, certain gut microbiota can enhance the effector cytokine production by the modification of their epigenetic signatures [122]. The gut microbiota can also affect cancer immune responses via the microbiota-derived metabolites [123]. Therefore, the role of the gut microbiome in carcinogenesis should be more precisely comprehended, and more accurate exploration in this field may be required.

Epigenetics with the adjustment of gut microbiota might be an advantageous modulator for novel cancer therapies. For example, it has been shown that combining PTT with an epigenetic therapy could provoke advantageous immunomodulatory responses that result in improved survival in a mice model, in which the epigenetic therapy could improve the host response to PTT by delaying the timing of tumor recurrence [124]. Similarly, it has also been shown that the epigenetic modification of tumor antigen levels may also be a novel approach to further enhance the effectiveness of the PDT cancer therapy [125]. Moreover, epigenetic treatments based on the administration of methyltransferase inhibitors in combination with the PDT therapy could offer further effective mechanisms leading to the considerable development of antitumor immunity with potentiated antitumor effects [126]. Other new modalities of targeted therapy and/or immunotherapy such as CAR-T-cell treatment, cancer vaccines, and/or oncolytic viral treatment could be also favorable with the appropriate alteration in the gut microbiome for the epigenetic modification, which could provide some extra weapons even against aggressive malignant tumors [127]. Probiotics and/or FMT have been found to be useful for the appropriate alteration in the gut microbiome in many diseases including cancer. However, they do not always seem to be safe [128,129]. Consideration of the risk/benefit ratio before setting these therapies should be recommended. Supervision is also mandatory to assess the security and effectiveness of the alteration in the gut microbiome [130]. For example, probiotics could be responsible for systemic infections including sepsis [131]. Some probiotic bacteria have also been recognized as spontaneous factors of endometritis, urinary tract infection, meningitis, and spleen abscess [132,133]. In addition, probiotic bacteria may occasionally result in chronic diarrhea, which might very faintly increase the risk of colorectal cancer [134]. Through the appropriate interference with commensal microflora, however, they could result in the decent performance in cancer therapies.

## 6. Future Perspectives

**Figure 3:**
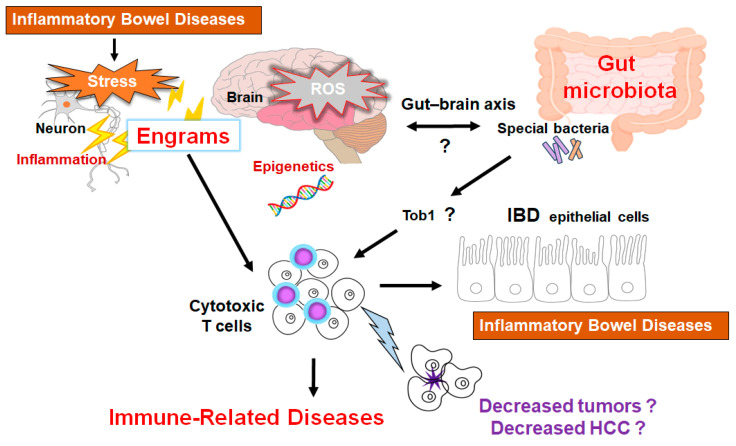
Schematic representation of the hypothetical effects of gut microbiota and/or brain engrams for the activation of cytotoxic T cells in the pathogenesis of inflammatory bowel diseases. Engrams may be shaped in the condition of repeated inflammation with ROS and/or various oxidative stresses, which could employ active immune cells to damage colon epithelium, brain neurons, and/or cancer cells. If certain commensal bacteria in the gut could inhibit the expression of Tob1, an APRO family protein, which might consequently further stimulate the cytotoxic T cells for the reduction of tumors. This concept could also be applied to the treatment of HCC. Note that some critical pathways such as Wnt/beta-catenin signaling have been omitted for clarity. “?” means for authors’ speculation.

Novel immunotherapies have turned out to be a breakthrough in inhibiting the growth of tumor cells by activating an antitumor immunity for various cancers, which has revolutionized treatment strategies and increased the chance of survival for various cancer patients [135]. Therefore, the treatment of HCC has also been drastically changing. These immunotherapies might considerably improve the survival of HCC patients even in advanced stages [136]. Moreover, this strategy could be further empowered by an adapted patient selection. Interestingly, it has been shown that gut microbiota-derived metabolites such as short-chain fatty acids (SCFAs) could be involved in the control of inflammation development, which might be associated with the immune power-shift for the cancer treatment efficacy [137]. Certain diets may be responsible for this beneficial modification of the gut microbiota which is an important factor for the therapeutic outcomes [138]. In particular, commensal bacteria are imperative in coordinating antitumor responses in the tumor microenvironment [139,140], which may be a key interface between cancer cells and anticancer T cells [141]. In this regard, we have reported an interesting relationship among the gut microbiota, cancer cells, and immune T cells by an “engram theory” for the innovative treatment of colon cancer within mice model (Figure 3) [67,142]. Further investigations are indeed required to understand the molecular mechanisms for developing superior treatment strategies and to obtain favorable clinical outcomes. Future investigation should focus on the design of patient-tailored cancer therapeutics by manipulating the diverse gut microbiota. 

Many studies have shown the important crosstalk between cancer cells and immune cells in a tumor microenvironment [143]. Various tumor cells have been shown to develop neovascularization by expressing various angiogenic factors for the progress of tumor growth in the tumor microenvironment [144]. Come to think of it, the development of cancers/tumors may depend on the function of host components such as gut microbiota, immune system, and/or tumor microenvironment with a close relationship each other. Again, a shift in mindset may be required for the concept of forthcoming cancer therapies. Probiotics may now be promising not only for cancer therapy, but also for the treatment of neurodegenerative disorders [145]. Henceforth, we now believe that the application of probiotics and/or FMT could be expanded for the treatment of autoimmune diseases and/or the related inflammatory diseases. Many researchers need to be united to comprehend the molecular mechanisms as well as to obtain some clues of therapeutic intervention against those intractable diseases including HCC. Remarkably, the marked distinctions in dysbiosis among patients with the HCC instigation imply that the restoration of microbial balance could yield substantive benefits in mitigating the pathology impeding their progression (Figure 3) [146,147].

## 7. Conclusions

The association between the immune system and gut microbiome could determine the effectiveness of several cancer therapies. Therefore, the appropriate combination of *probiotics* with novel cancer chemotherapies may also improve the therapeutic consequence of patients suffering from human HCC.

## Abbreviations

ALD	alcohol-associated liver disease
CAR	chimeric antigen receptor
CTLA4	cytotoxic T-lymphocyte-associated protein 4
FMT	fecal microbiota transplantation
HBV	hepatitis B virus
HCV	hepatitis C virus
HCC	hepatocellular carcinoma
LPS	lipopolysaccharide
MASH	metabolic dysfunction associated steatohepatitis
MASLD	metabolic dysfunction associated steatotic liver disease
MHC	major histocompatibility complex
PD-1	programmed cell death protein-1
PD-L1	programmed cell death protein ligand-1
PDT	photodynamic therapy
PTT	photothermal therapy
ROS	reactive oxygen species
SCFAs	short-chain fatty acids
STAT	signal transducer and activator of transcription
Th17	T helper 17 cell
TLRs	toll-like receptors
Treg	regulatory T cell
VEGF	vascular endothelial growth factor A

## References

[1] Bray F., Ferlay J., Soerjomataram I., Siegel R.L., Torre L.A., Jemal A. (2018). Global cancer statistics 2018: GLOBOCAN estimates of incidence and mortality worldwide for 36 cancers in 185 countries. CA Cancer J. Clin..

[2] Canale M., Ulivi P., Foschi F.G., Scarpi E., De Matteis S., Donati G., Ercolani G., Scartozzi M., Faloppi L., Passardi A. (2018). Clinical and circulating biomarkers of survival and recurrence after radiofrequency ablation in patients with hepatocellular carcinoma. Crit. Rev. Oncol. Hematol..

[3] Choi J.W., Lee J.M., Lee D.H., Yoon J.H., Kim Y.J., Lee J.H., Yu S.J., Cho E.J. (2020). Radiofrequency ablation using internally cooled wet electrodes in bipolar mode for the treatment of recurrent hepatocellular carcinoma after locoregional treatment: A randomized prospective comparative study. PLoS ONE.

[4] Ilagan C.H., Goldman D.A., Gönen M., Aveson V.G., Babicky M., Balachandran V.P., Drebin J.A., Jarnagin W.R., Wei A.C., Kingham T.P. (2022). Recurrence of Hepatocellular Carcinoma After Complete Radiologic Response to Trans-Arterial Embolization: A Retrospective Study on Patterns, Treatments, and Prognoses. Ann. Surg. Oncol..

[5] Bruix J., Qin S., Merle P., Granito A., Huang Y.H., Bodoky G., Pracht M., Yokosuka O., Rosmorduc O., Breder V. (2017). Regorafenib for patients with hepatocellular carcinoma who progressed on sorafenib treatment (RESORCE): A randomised, double-blind, placebo-controlled, phase 3 trial. Lancet.

[6] Hu W.Y., Wei H.Y., Li K.M., Wang R.B., Xu X.Q., Feng R. (2020). LINC00511 as a ceRNA promotes cell malignant behaviors and correlates with prognosis of hepatocellular carcinoma patients by modulating miR-195/EYA1 axis. Biomed. Pharmacother..

[7] Ma C., Kesarwala A.H., Eggert T., Medina-Echeverz J., Kleiner D.E., Jin P., Stroncek D.F., Terabe M., Kapoor V., ElGindi M. (2016). NAFLD causes selective CD4(+) T lymphocyte loss and promotes hepatocarcinogenesis. Nature.

[8] Mehal W.Z. (2013). The Gordian Knot of dysbiosis, obesity and NAFLD. Nat. Rev. Gastroenterol. Hepatol..

[9] Villanueva A. (2019). Hepatocellular Carcinoma. N. Engl. J. Med..

[10] Chen W., Desert R., Ge X., Han H., Song Z., Das S., Athavale D., You H., Nieto N. (2021). The Matrisome Genes From Hepatitis B-Related Hepatocellular Carcinoma Unveiled. Hepatol. Commun..

[11] Quesada-Vázquez S., Bone C., Saha S., Triguero I., Colom-Pellicer M., Aragonès G., Hildebrand F., Del Bas J.M., Caimari A., Beraza N. (2022). Microbiota Dysbiosis and Gut Barrier Dysfunction Associated with Non-Alcoholic Fatty Liver Disease Are Modulated by a Specific Metabolic Cofactors’ Combination. Int. J. Mol. Sci..

[12] Guo X., Okpara E.S., Hu W., Yan C., Wang Y., Liang Q., Chiang J.Y.L., Han S. (2022). Interactive Relationships between Intestinal Flora and Bile Acids. Int. J. Mol. Sci..

[13] Tohme S., Simmons R.L., Tsung A. (2017). Surgery for Cancer: A Trigger for Metastases. Cancer Res..

[14] Goldstein M.R., Mascitelli L. (2011). Surgery and cancer promotion: Are we trading beauty for cancer?. QJM.

[15] Shaverdian N., Lisberg A.E., Bornazyan K., Veruttipong D., Goldman J.W., Formenti S.C., Garon E.B., Lee P. (2017). Previous radiotherapy and the clinical activity and toxicity of pembrolizumab in the treatment of non-small-cell lung cancer: A secondary analysis of the KEYNOTE-001 phase 1 trial. Lancet Oncol..

[16] Seiwert T.Y., Kiess A.P. (2021). Time to Debunk an Urban Myth? The “Abscopal Effect” with Radiation and Anti-PD-1. J. Clin. Oncol..

[17] Chen D., Verma V., Patel R.R., Barsoumian H.B., Cortez M.A., Welsh J.W. (2020). Absolute Lymphocyte Count Predicts Abscopal Responses and Outcomes in Patients Receiving Combined Immunotherapy and Radiation Therapy: Analysis of 3 Phase 1/2 Trials. Int. J. Radiat. Oncol. Biol. Phys..

[18] Zhai D., An D., Wan C., Yang K. (2022). Radiotherapy: Brightness and darkness in the era of immunotherapy. Transl. Oncol..

[19] Terasaki Y., Ohsawa I., Terasaki M., Takahashi M., Kunugi S., Dedong K., Urushiyama H., Amenomori S., Kaneko-Togashi M., Kuwahara N. (2011). Hydrogen therapy attenuates irradiation-induced lung damage by reducing oxidative stress. Am. J. Physiol. Lung Cell Mol. Physiol..

[20] Lundgren S., Karnevi E., Elebro J., Nodin B., Karlsson M.C.I., Eberhard J., Leandersson K., Jirström K. (2017). The clinical importance of tumour-infiltrating macrophages and dendritic cells in periampullary adenocarcinoma differs by morphological subtype. J. Transl. Med..

[21] Ryter S.W., Kim H.P., Hoetzel A., Park J.W., Nakahira K., Wang X., Choi A.M. (2007). Mechanisms of cell death in oxidative stress. Antioxid. Redox Signal..

[22] Siegel R.J., Singh A.K., Panipinto P.M., Shaikh F.S., Vinh J., Han S.U., Kenney H.M., Schwarz E.M., Crowson C.S., Khuder S.A. (2022). Extracellular sulfatase-2 is overexpressed in rheumatoid arthritis and mediates the TNF-α-induced inflammatory activation of synovial fibroblasts. Cell Mol. Immunol..

[23] Raguz S., Yagüe E. (2008). Resistance to chemotherapy: New treatments and novel insights into an old problem. Br. J. Cancer.

[24] Choy H., Kim D.W. (2003). Chemotherapy and irradiation interaction. Semin. Oncol..

[25] McGuigan A., Kelly P., Turkington R.C., Jones C., Coleman H.G., McCain R.S. (2018). Pancreatic cancer: A review of clinical diagnosis, epidemiology, treatment and outcomes. World J. Gastroenterol..

[26] Jung H.S., Verwilst P., Sharma A., Shin J., Sessler J.L., Kim J.S. (2018). Organic molecule-based photothermal agents: An expanding photothermal therapy universe. Chem. Soc. Rev..

[27] Fan Z., Zhuang C., Wang S., Zhang Y. (2021). Photodynamic and Photothermal Therapy of Hepatocellular Carcinoma. Front. Oncol..

[28] Li X., Lovell J.F., Yoon J., Chen X. (2020). Clinical development and potential of photothermal and photodynamic therapies for cancer. Nat. Rev. Clin. Oncol..

[29] Dun X., Liu S., Ge N., Liu M., Li M., Zhang J., Bao H., Li B., Zhang H., Cui L. (2022). Photothermal effects of CuS-BSA nanoparticles on H22 hepatoma-bearing mice. Front. Pharmacol..

[30] Casini A., Leone S., Vaccaro R., Vivacqua G., Ceci L., Pannarale L., Franchitto A., Onori P., Gaudio E., Mancinelli R. (2022). The Emerging Role of Ferroptosis in Liver Cancers. Life.

[31] Kaufmann S.H.E., Dorhoi A., Hotchkiss R.S., Bartenschlager R. (2018). Host-directed therapies for bacterial and viral infections. Nat. Rev. Drug Discov..

[32] Dyck L., Mills K.H.G. (2017). Immune checkpoints and their inhibition in cancer and infectious diseases. Eur. J. Immunol..

[33] Wei S.C., Duffy C.R., Allison J.P. (2018). Fundamental Mechanisms of Immune Checkpoint Blockade Therapy. Cancer Discov..

[34] Ribas A., Wolchok J.D. (2018). Cancer immunotherapy using checkpoint blockade. Science.

[35] Korman A.J., Peggs K.S., Allison J.P. (2006). Checkpoint blockade in cancer immunotherapy. Adv. Immunol..

[36] Bagchi S., Yuan R., Engleman E.G. (2021). Immune Checkpoint Inhibitors for the Treatment of Cancer: Clinical Impact and Mechanisms of Response and Resistance. Annu. Rev. Pathol..

[37] Andrews L.P., Yano H., Vignali D.A.A. (2019). Inhibitory receptors and ligands beyond PD-1, PD-L1 and CTLA-4: Breakthroughs or backups. Nat. Immunol..

[38] Donisi C., Puzzoni M., Ziranu P., Lai E., Mariani S., Saba G., Impera V., Dubois M., Persano M., Migliari M. (2021). Immune Checkpoint Inhibitors in the Treatment of HCC. Front. Oncol..

[39] Lai E., Astara G., Ziranu P., Pretta A., Migliari M., Dubois M., Donisi C., Mariani S., Liscia N., Impera V. (2021). Introducing immunotherapy for advanced hepatocellular carcinoma patients: Too early or too fast?. Crit. Rev. Oncol. Hematol..

[40] Greten T.F., Lai C.W., Li G., Staveley-O’Carroll K.F. (2019). Targeted and Immune-Based Therapies for Hepatocellular Carcinoma. Gastroenterology.

[41] Katariya N.N., Lizaola-Mayo B.C., Chascsa D.M., Giorgakis E., Aqel B.A., Moss A.A., Uson Junior P.L.S., Borad M.J., Mathur A.K. (2022). Immune Checkpoint Inhibitors as Therapy to Down-Stage Hepatocellular Carcinoma Prior to Liver Transplantation. Cancers.

[42] Abdelrahim M., Esmail A., Saharia A., Abudayyeh A., Abdel-Wahab N., Diab A., Murakami N., Kaseb A.O., Chang J.C., Gaber A.O. (2022). Utilization of Immunotherapy for the Treatment of Hepatocellular Carcinoma in the Peri-Transplant Setting: Transplant Oncology View. Cancers.

[43] Hewitt D.B., Rahnemai-Azar A.A., Pawlik T.M. (2021). Potential experimental immune checkpoint inhibitors for the treatment of cancer of the liver. Expert. Opin. Investig. Drugs.

[44] Zhang Y., Liu Z., Tian M., Hu X., Wang L., Ji J., Liao A. (2018). The altered PD-1/PD-L1 pathway delivers the ’one-two punch’ effects to promote the Treg/Th17 imbalance in pre-eclampsia. Cell Mol. Immunol..

[45] Kim S.T., Chu Y., Misoi M., Suarez-Almazor M.E., Tayar J.H., Lu H., Buni M., Kramer J., Rodriguez E., Hussain Z. (2022). Distinct molecular and immune hallmarks of inflammatory arthritis induced by immune checkpoint inhibitors for cancer therapy. Nat. Commun..

[46] Okiyama N., Tanaka R. (2022). Immune-related adverse events in various organs caused by immune checkpoint inhibitors. Allergol. Int..

[47] Li S., Na R., Li X., Zhang Y., Zheng T. (2022). Targeting interleukin-17 enhances tumor response to immune checkpoint inhibitors in colorectal cancer. Biochim. Biophys. Acta Rev. Cancer.

[48] Ramesh R., Kozhaya L., McKevitt K., Djuretic I.M., Carlson T.J., Quintero M.A., McCauley J.L., Abreu M.T., Unutmaz D., Sundrud M.S. (2014). Pro-inflammatory human Th17 cells selectively express P-glycoprotein and are refractory to glucocorticoids. J. Exp. Med..

[49] Melin A., Routier É., Roy S., Pradere P., Le Pavec J., Pierre T., Chanson N., Scoazec J.Y., Lambotte O., Robert C. (2022). Sarcoid-like Granulomatosis Associated with Immune Checkpoint Inhibitors in Melanoma. Cancers.

[50] Downs-Canner S., Berkey S., Delgoffe G.M., Edwards R.P., Curiel T., Odunsi K., Bartlett D.L., Obermajer N. (2017). Suppressive IL-17A^+^Foxp3^+^ and ex-Th17 IL-17A^neg^Foxp3^+^ T_reg_ cells are a source of tumour-associated T_reg_ cells. Nat. Commun..

[51] Saenz S.A., Local A., Carr T., Shakya A., Koul S., Hu H., Chourb L., Stedman J., Malley J., D’Agostino L.A. (2021). Small molecule allosteric inhibitors of RORγt block Th17-dependent inflammation and associated gene expression in vivo. PLoS ONE.

[52] Kendall T., Verheij J., Gaudio E., Evert M., Guido M., Goeppert B., Carpino G. (2019). Anatomical, histomorphological and molecular classification of cholangiocarcinoma. Liver Int..

[53] Yoo H.J., Harapan B.N. (2021). Chimeric antigen receptor (CAR) immunotherapy: Basic principles, current advances, and future prospects in neuro-oncology. Immunol. Res..

[54] Huang R., Wang X., Zhang X. (2022). Unity brings strength: Combination of CAR-T cell therapy and HSCT. Cancer Lett..

[55] Chung H., Jung H., Noh J.Y. (2021). Emerging Approaches for Solid Tumor Treatment Using CAR-T Cell Therapy. Int. J. Mol. Sci..

[56] Sun B., Yang D., Dai H., Liu X., Jia R., Cui X., Li W., Cai C., Xu J., Zhao X. (2019). Eradication of Hepatocellular Carcinoma by NKG2D-Based CAR-T Cells. Cancer Immunol. Res..

[57] Jose A., Bavetta M.G., Martinelli E., Bronte F., Giunta E.F., Manu K.A. (2022). Hepatocellular Carcinoma: Current Therapeutic Algorithm for Localized and Advanced Disease. J. Oncol..

[58] Howells A., Marelli G., Lemoine N.R., Wang Y. (2017). Oncolytic Viruses-Interaction of Virus and Tumor Cells in the Battle to Eliminate Cancer. Front. Oncol..

[59] Luo C., Wang P., He S., Zhu J., Shi Y., Wang J. (2022). Progress and Prospect of Immunotherapy for Triple-Negative Breast Cancer. Front. Oncol..

[60] Zhou Y., Wang Q., Ying Q., Zhang X., Chen K., Ye T., Li G. (2023). Effects of Oncolytic Vaccinia Viruses Harboring Different Marine Lectins on Hepatocellular Carcinoma Cells. Int. J. Mol. Sci..

[61] Zheng X., Xu W., Ying Q., Ni J., Jia X., Zhou Y., Ye T., Li G., Chen K. (2022). Oncolytic Vaccinia Virus Carrying *Aphrocallistes vastus* Lectin (oncoVV-AVL) Enhances Inflammatory Response in Hepatocellular Carcinoma Cells. Mar. Drugs.

[62] Qi X., Liu Y., Hussein S., Choi G., Kimchi E.T., Staveley-O’Carroll K.F., Li G. (2022). The Species of Gut Bacteria Associated with Antitumor Immunity in Cancer Therapy. Cells.

[63] Wang Z., Li L., Wang S., Wei J., Qu L., Pan L., Xu K. (2022). The role of the gut microbiota and probiotics associated with microbial metabolisms in cancer prevention and therapy. Front. Pharmacol..

[64] Luo M., Chen X., Gao H., Yang F., Chen J., Qiao Y. (2022). Bacteria-mediated cancer therapy: A versatile bio-sapper with translational potential. Front. Oncol..

[65] Nejman D., Livyatan I., Fuks G., Gavert N., Zwang Y., Geller L.T., Rotter-Maskowitz A., Weiser R., Mallel G., Gigi E. (2020). The human tumor microbiome is composed of tumor type-specific intracellular bacteria. Scinence.

[66] Kaźmierczak-Siedlecka K., Daca A., Fic M., van de Wetering T., Folwarski M., Makarewicz W. (2020). Therapeutic methods of gut microbiota modification in colorectal cancer management—Fecal microbiota transplantation, prebiotics, probiotics, and synbiotics. Gut Microbes.

[67] Ikeda Y., Taniguchi K., Yoshikawa S., Sawamura H., Tsuji A., Matsuda S. (2022). A budding concept with certain microbiota, anti-proliferative family proteins, and engram theory for the innovative treatment of colon cancer. Explor. Med..

[68] Wang C., Li W., Wang H., Ma Y., Zhao X., Zhang X., Yang H., Qian J., Li J. (2019). Saccharomyces boulardii alleviates ulcerative colitis carcinogenesis in mice by reducing TNF-α and IL-6 levels and functions and by rebalancing intestinal microbiota. BMC Microbiol..

[69] Shi Y., Xu L.Z., Peng K., Wu W., Wu R., Liu Z.Q., Yang G., Geng X.R., Liu J., Liu Z.G. (2015). Specific immunotherapy in combination with Clostridium butyricum inhibits allergic inflammation in the mouse intestine. Sci. Rep..

[70] Uchugonova A., Zhang Y., Salz R., Liu F., Suetsugu A., Zhang L., Koenig K., Hoffman R.M., Zhao M. (2015). Imaging the Different Mechanisms of Prostate Cancer Cell-killing by Tumor-targeting Salmonella typhimurium A1-R. Anticancer Res..

[71] Li L., You L.S., Mao L.P., Jin S.H., Chen X.H., Qian W.B. (2018). Combing oncolytic adenovirus expressing Beclin-1 with chemotherapy agent doxorubicin synergistically enhances cytotoxicity in human CML cells in vitro. Acta Pharmacol. Sin..

[72] Wang X., Liu Z., Sui X., Wu Q., Wang J., Xu C. (2019). Elemene injection as adjunctive treatment to platinum-based chemotherapy in patients with stage III/IV non-small cell lung cancer: A meta-analysis following the PRISMA guidelines. Phytomedicine.

[73] Din M.O., Danino T., Prindle A., Skalak M., Selimkhanov J., Allen K., Julio E., Atolia E., Tsimring L.S., Bhatia S.N. (2016). Synchronized cycles of bacterial lysis for in vivo delivery. Nature.

[74] McNerney M.P., Doiron K.E., Ng T.L., Chang T.Z., Silver P.A. (2021). Theranostic cells: Emerging clinical applications of synthetic biology. Nat. Rev. Genet..

[75] Lu Y., Luo X., Yang D., Li Y., Gong T., Li B., Cheng J., Chen R., Guo X., Yuan W. (2022). Effects of probiotic supplementation on related side effects after chemoradiotherapy in cancer patients. Front. Oncol..

[76] Burdelya L.G., Krivokrysenko V.I., Tallant T.C., Strom E., Gleiberman A.S., Gupta D., Kurnasov O.V., Fort F.L., Osterman A.L., Didonato J.A. (2008). An agonist of toll-like receptor 5 has radioprotective activity in mouse and primate models. Science.

[77] Abdollahi H. (2015). Beneficial effects of cellular autofluorescence following ionization radiation: Hypothetical approaches for radiation protection and enhancing radiotherapy effectiveness. Med. Hypotheses.

[78] Bettegowda C., Dang L.H., Abrams R., Huso D.L., Dillehay L., Cheong I., Agrawal N., Borzillary S., McCaffery J.M., Watson E.L. (2003). Overcoming the hypoxic barrier to radiation therapy with anaerobic bacteria. Proc. Natl. Acad. Sci. USA.

[79] Poonacha K.N.T., Villa T.G., Notario V. (2022). The Interplay among Radiation Therapy, Antibiotics and the Microbiota: Impact on Cancer Treatment Outcomes. Antibiotics.

[80] Lee S.H., Cho S.Y., Yoon Y., Park C., Sohn J., Jeong J.J., Jeon B.N., Jang M., An C., Lee S. (2021). Bifidobacterium bifidum strains synergize with immune checkpoint inhibitors to reduce tumour burden in mice. Nat. Microbiol..

[81] Zhuo Q., Yu B., Zhou J., Zhang J., Zhang R., Xie J., Wang Q., Zhao S. (2019). Lysates of Lactobacillus acidophilus combined with CTLA-4-blocking antibodies enhance antitumor immunity in a mouse colon cancer model. Sci. Rep..

[82] Ahmadi Badi S., Moshiri A., Ettehad Marvasti F., Mojtahedzadeh M., Kazemi V., Siadat S.D. (2020). Extraction and Evaluation of Outer Membrane Vesicles from Two Important Gut Microbiota Members, Bacteroides fragilis and Bacteroides thetaiotaomicron. Cell J..

[83] Vétizou M., Pitt J.M., Daillère R., Lepage P., Waldschmitt N., Flament C., Rusakiewicz S., Routy B., Roberti M.P., Duong C.P. (2015). Anticancer immunotherapy by CTLA-4 blockade relies on the gut microbiota. Science.

[84] Zhang M., Qiu X., Zhang H., Yang X., Hong N., Yang Y., Chen H., Yu C. (2014). *Faecalibacterium prausnitzii* inhibits interleukin-17 to ameliorate colorectal colitis in rats. PLoS ONE.

[85] Zhou L., Zhang M., Wang Y., Dorfman R.G., Liu H., Yu T., Chen X., Tang D., Xu L., Yin Y. (2018). *Faecalibacterium prausnitzii* Produces Butyrate to Maintain Th17/Treg Balance and to Ameliorate Colorectal Colitis by Inhibiting Histone Deacetylase. Inflamm. Bowel Dis..

[86] Kaźmierczak-Siedlecka K., Skonieczna-Żydecka K., Hupp T., Duchnowska R., Marek-Trzonkowska N., Połom K. (2022). Next-generation probiotics—Do they open new therapeutic strategies for cancer patients?. Gut Microbes.

[87] Behary J., Raposo A.E., Amorim N.M.L., Zheng H., Gong L., McGovern E., Chen J., Liu K., Beretov J., Theocharous C. (2021). Defining the temporal evolution of gut dysbiosis and inflammatory responses leading to hepatocellular carcinoma in Mdr2 −/− mouse model. BMC Microbiol..

[88] Ponziani F.R., Bhoori S., Castelli C., Putignani L., Rivoltini L., Del Chierico F., Sanguinetti M., Morelli D., Paroni Sterbini F., Petito V. (2019). Hepatocellular Carcinoma Is Associated With Gut Microbiota Profile and Inflammation in Nonalcoholic Fatty Liver Disease. Hepatology.

[89] Zhang X., Coker O.O., Chu E.S., Fu K., Lau H.C.H., Wang Y.X., Chan A.W.H., Wei H., Yang X., Sung J.J.Y. (2021). Dietary cholesterol drives fatty liver-associated liver cancer by modulating gut microbiota and metabolites. Gut.

[90] Rezasoltani S., Yadegar A., Asadzadeh Aghdaei H., Reza Zali M. (2021). Modulatory effects of gut microbiome in cancer immunotherapy: A novel paradigm for blockade of immune checkpoint inhibitors. Cancer Med..

[91] Wu Y., Zheng L. (2012). Dynamic education of macrophages in different areas of human tumors. Cancer Microenviron..

[92] Ahmed F., Steele J.C., Herbert J.M., Steven N.M., Bicknell R. (2008). Tumor stroma as a target in cancer. Curr. Cancer Drug Targets.

[93] Zhang J.P., Yan J., Xu J., Pang X.H., Chen M.S., Li L., Wu C., Li S.P., Zheng L. (2009). Increased intratumoral IL-17-producing cells correlate with poor survival in hepatocellular carcinoma patients. J. Hepatol..

[94] Zhao F., Hoechst B., Gamrekelashvili J., Ormandy L.A., Voigtländer T., Wedemeyer H., Ylaya K., Wang X.W., Hewitt S.M., Manns M.P. (2012). Human CCR4+ CCR6+ Th17 cells suppress autologous CD8+ T cell responses. J. Immunol..

[95] Liao R., Sun J., Wu H., Yi Y., Wang J.X., He H.W., Cai X.Y., Zhou J., Cheng Y.F., Fan J. (2013). High expression of IL-17 and IL-17RE associate with poor prognosis of hepatocellular carcinoma. J. Exp. Clin. Cancer Res..

[96] Kuang D.M., Peng C., Zhao Q., Wu Y., Chen M.S., Zheng L. (2010). Activated monocytes in peritumoral stroma of hepatocellular carcinoma promote expansion of memory T helper 17 cells. Hepatology.

[97] Michaelis L., Treß M., Löw H.C., Klees J., Klameth C., Lange A., Grießhammer A., Schäfer A., Menz S., Steimle A. (2021). Gut Commensal-Induced IκBζ Expression in Dendritic Cells Influences the Th17 Response. Front. Immunol..

[98] Sung C.Y., Lee N.P., El-Nezami H. (2012). Regulation of T helper 17 by bacteria: An approach for the treatment of hepatocellular carcinoma. Int. J. Hepatol..

[99] Gu F.M., Li Q.L., Gao Q., Jiang J.H., Zhu K., Huang X.Y., Pan J.F., Yan J., Hu J.H., Wang Z. (2011). IL-17 induces AKT-dependent IL-6/JAK2/STAT3 activation and tumor progression in hepatocellular carcinoma. Mol. Cancer.

[100] Qin H., Yuan B., Huang W., Wang Y. (2022). Utilizing Gut Microbiota to Improve Hepatobiliary Tumor Treatments: Recent Advances. Front. Oncol..

[101] Mikó E., Vida A., Bai P. (2016). Translational aspects of the microbiome-to be exploited. Cell Biol. Toxicol..

[102] Kovács T., Mikó E., Vida A., Sebő É., Toth J., Csonka T., Boratkó A., Ujlaki G., Lente G., Kovács P. (2019). Cadaverine, a metabolite of the microbiome, reduces breast cancer aggressiveness through trace amino acid receptors. Sci. Rep..

[103] Yu L.X., Schwabe R.F. (2017). The gut microbiome and liver cancer: Mechanisms and clinical translation. Nat. Rev. Gastroenterol. Hepatol..

[104] Yuan J., Chen C., Cui J., Lu J., Yan C., Wei X., Zhao X., Li N., Li S., Xue G. (2019). Fatty Liver Disease Caused by High-Alcohol-Producing Klebsiella pneumoniae. Cell Metab..

[105] Lapidot Y., Amir A., Nosenko R., Uzan-Yulzari A., Veitsman E., Cohen-Ezra O., Davidov Y., Weiss P., Bradichevski T., Segev S. (2020). Alterations in the Gut Microbiome in the Progression of Cirrhosis to Hepatocellular Carcinoma. mSystems.

[106] Ma C., Han M., Heinrich B., Fu Q., Zhang Q., Sandhu M., Agdashian D., Terabe M., Berzofsky J.A., Fako V. (2018). Gut microbiome-mediated bile acid metabolism regulates liver cancer via NKT cells. Science.

[107] Routy B., Le Chatelier E., Derosa L., Duong C.P.M., Alou M.T., Daillère R., Fluckiger A., Messaoudene M., Rauber C., Roberti M.P. (2018). Gut microbiome influences efficacy of PD-1-based immunotherapy against epithelial tumors. Science.

[108] Gopalakrishnan V., Spencer C.N., Nezi L., Reuben A., Andrews M.C., Karpinets T.V., Prieto P.A., Vicente D., Hoffman K., Wei S.C. (2018). Gut microbiome modulates response to anti-PD-1 immunotherapy in melanoma patients. Science.

[109] Matson V., Fessler J., Bao R., Chongsuwat T., Zha Y., Alegre M.L., Luke J.J., Gajewski T.F. (2018). The commensal microbiome is associated with anti-PD-1 efficacy in metastatic melanoma patients. Science.

[110] Yoshimoto S., Loo T.M., Atarashi K., Kanda H., Sato S., Oyadomari S., Iwakura Y., Oshima K., Morita H., Hattori M. (2013). Obesity-induced gut microbial metabolite promotes liver cancer through senescence secretome. Nature.

[111] Dapito D.H., Mencin A., Gwak G.Y., Pradere J.P., Jang M.K., Mederacke I., Caviglia J.M., Khiabanian H., Adeyemi A., Bataller R. (2012). Promotion of hepatocellular carcinoma by the intestinal microbiota and TLR. Cancer Cell.

[112] Zheng Y., Wang T., Tu X., Huang Y., Zhang H., Tan D., Jiang W., Cai S., Zhao P., Song R. (2019). Gut microbiome affects the response to anti-PD-1 immunotherapy in patients with hepatocellular carcinoma. J. Immunother. Cancer.

[113] Fernandes M.R., Aggarwal P., Costa R.G.F., Cole A.M., Trinchieri G. (2022). Targeting the gut microbiota for cancer therapy. Nat. Rev. Cancer.

[114] Dai E., Zhu Z., Wahed S., Qu Z., Storkus W.J., Guo Z.S. (2021). Epigenetic modulation of antitumor immunity for improved cancer immunotherapy. Mol. Cancer.

[115] Burr M.L., Sparbier C.E., Chan K.L., Chan Y.C., Kersbergen A., Lam E.Y.N., Azidis-Yates E., Vassiliadis D., Bell C.C., Gilan O. (2019). An evolutionarily conserved function of polycomb silences the MHC class I antigen presentation pathway and enables immune evasion in cancer. Cancer Cell.

[116] Greger V., Passarge E., Höpping W., Messmer E., Horsthemke B. (1989). Epigenetic changes may contribute to the formation and spontaneous regression of retinoblastoma. Hum. Genet..

[117] Saito Y., Liang G., Egger G., Friedman J.M., Chuang J.C., Coetzee G.A., Jones P.A. (2006). Specific activation of microRNA-127 with downregulation of the proto-oncogene BCL6 by chromatin-modifying drugs in human cancer cells. Cancer Cell.

[118] Senga S.S., Grose R.P. (2021). Hallmarks of cancer-the new testament. Open Biol..

[119] Wang G., He X., Wang Q. (2023). Intratumoral bacteria are an important “accomplice” in tumor development and metastasis. Biochim. Biophys. Acta Rev. Cancer.

[120] Yang F., Yang Y., Chen L., Zhang Z., Liu L., Zhang C., Mai Q., Chen Y., Chen Z., Lin T. (2022). The gut microbiota mediates protective immunity against tuberculosis via modulation of lncRNA. Gut Microbes.

[121] Niller H.H., Minarovits J. (2016). Patho-epigenetics of Infectious Diseases Caused by Intracellular Bacteria. Adv. Exp. Med. Biol..

[122] Luu M., Schütz B., Lauth M., Visekruna A. (2023). The Impact of Gut Microbiota-Derived Metabolites on the Tumor Immune Microenvironment. Cancers.

[123] Cerf-Bensussan N., Gaboriau-Routhiau V. (2010). The immune system and the gut microbiota: Friends or foes?. Nat. Rev. Immunol..

[124] Ledezma D.K., Balakrishnan P.B., Cano-Mejia J., Sweeney E.E., Hadley M., Bollard C.M., Villagra A., Fernandes R. (2020). Indocyanine Green-Nexturastat A-PLGA Nanoparticles Combine Photothermal and Epigenetic Therapy for Melanoma. Nanomaterials.

[125] Wachowska M., Gabrysiak M., Muchowicz A., Bednarek W., Barankiewicz J., Rygiel T., Boon L., Mroz P., Hamblin M.R., Golab J. (2014). 5-Aza-2′-deoxycytidine potentiates antitumour immune response induced by photodynamic therapy. Eur. J. Cancer.

[126] Wachowska M., Muchowicz A., Golab J. (2015). Targeting Epigenetic Processes in Photodynamic Therapy-Induced Anticancer Immunity. Front. Oncol..

[127] Trifylli E.M., Koustas E., Papadopoulos N., Sarantis P., Aloizos G., Damaskos C., Garmpis N., Garmpi A., Karamouzis M.V. (2022). An Insight into the Novel Immunotherapy and Targeted Therapeutic Strategies for Hepatocellular Carcinoma and Cholangiocarcinoma. Life.

[128] Hill C., Guarner F., Reid G., Gibson G.R., Merenstein D.J., Pot B., Morelli L., Canani R.B., Flint H.J., Salminen S. (2014). Expert consensus document. The International Scientific Association for Probiotics and Prebiotics consensus statement on the scope and appropriate use of the term probiotic. Nat. Rev. Gastroenterol. Hepatol..

[129] Pace F., Macchini F., Castagna V.M. (2020). Safety of probiotics in humans: A dark side revealed?. Dig. Liver Dis..

[130] Wallace C., Sinopoulou V., Gordon M., Akobeng A.K., Llanos-Chea A., Hungria G., Febo-Rodriguez L., Fifi A., Fernandez Valdes L., Langshaw A. (2022). Probiotics for treatment of chronic constipation in children. Cochrane Database Syst. Rev..

[131] Sun F., Zhang Q., Zhao J., Zhang H., Zhai Q., Chen W. (2019). A potential species of next-generation probiotics? The dark and light sides of Bacteroides fragilis in health. Food Res. Int..

[132] Bayer A.S., Chow A.W., Betts D., Guze L.B. (1978). Lactobacillemia--report of nine cases. Important clinical and therapeutic considerations. Am. J. Med..

[133] Dickgiesser U., Weiss N., Fritsche D. (1984). Lactobacillus gasseri as the cause of septic urinary infection. Infection.

[134] Tan K.P., Yang M., Ito S. (2007). Activation of nuclear factor (erythroid-2 like) factor 2 by toxic bile acids provokes adaptive defense responses to enhance cell survival at the emergence of oxidative stress. Mol. Pharmacol..

[135] Hiam-Galvez K.J., Allen B.M., Spitzer M.H. (2021). Systemic immunity in cancer. Nat. Rev. Cancer.

[136] Reig M., Forner A., Rimola J., Ferrer-Fàbrega J., Burrel M., Garcia-Criado Á., Kelley R.K., Galle P.R., Mazzaferro V., Salem R. (2022). BCLC strategy for prognosis prediction and treatment recommendation: The 2022 update. J. Hepatol..

[137] Jiang X., Zheng J., Zhang S., Wang B., Wu C., Guo X. (2020). Advances in the Involvement of Gut Microbiota in Pathophysiology of NAFLD. Front. Med..

[138] Alexander J.L., Wilson I.D., Teare J., Marchesi J.R., Nicholson J.K., Kinross J.M. (2017). Gut microbiota modulation of chemotherapy efficacy and toxicity. Nat. Rev. Gastroenterol. Hepatol..

[139] Mukaida N. (2014). Intestinal microbiota: Unexpected alliance with tumor therapy. Immunotherapy.

[140] Iida N., Dzutsev A., Stewart C.A., Smith L., Bouladoux N., Weingarten R.A., Molina D.A., Salcedo R., Back T., Cramer S. (2013). Commensal bacteria control cancer response to therapy by modulating the tumor microenvironment. Science.

[141] Pagadala M., Sears T.J., Wu V.H., Pérez-Guijarro E., Kim H., Castro A., Talwar J.V., Gonzalez-Colin C., Cao S., Schmiedel B.J. (2023). Germline modifiers of the tumor immune microenvironment implicate drivers of cancer risk and immunotherapy response. Nat. Commun..

[142] Yoshikawa S., Taniguchi K., Sawamura H., Ikeda Y., Tsuji A., Matsuda S. (2022). Encouraging probiotics for the prevention and treatment of immune-related adverse events in novel immunotherapies against malignant glioma. Explor. Target. Antitumor Ther..

[143] Ayob A.Z., Ramasamy T.S. (2018). Cancer stem cells as key drivers of tumour progression. J. Biomed. Sci..

[144] Garza Treviño E.N., González P.D., Valencia Salgado C.I., Martinez Garza A. (2019). Effects of pericytes and colon cancer stem cells in the tumor microenvironment. Cancer Cell Int..

[145] Yoshikawa S., Taniguchi K., Sawamura H., Ikeda Y., Tsuji A., Matsuda S. (2022). A New Concept of Associations between Gut Microbiota, Immunity and Central Nervous System for the Innovative Treatment of Neurodegenerative Disorders. Metabolites.

[146] Abenavoli L., Maurizi V., Rinninella E., Tack J., Di Berardino A., Santori P., Rasetti C., Procopio A.C., Boccuto L., Scarpellini E. (2022). Fecal Microbiota Transplantation in NAFLD Treatment. Medicina.

[147] Boursier J., Mueller O., Barret M., Machado M., Fizanne L., Araujo-Perez F., Guy C.D., Seed P.C., Rawls J.F., David L.A. (2016). The Severity of Nonalcoholic Fatty Liver Disease Is Associated with Gut Dysbiosis and Shift in the Metabolic Function of the Gut Microbiota. Hepatology.

